# Anti-Caries Effects of Dental Adhesives Containing Quaternary Ammonium Methacrylates with Different Chain Lengths

**DOI:** 10.3390/ma10060643

**Published:** 2017-06-12

**Authors:** Qi Han, Bolei Li, Xuedong Zhou, Yang Ge, Suping Wang, Mingyun Li, Biao Ren, Haohao Wang, Keke Zhang, Hockin H. K. Xu, Xian Peng, Mingye Feng, Michael D. Weir, Yu Chen, Lei Cheng

**Affiliations:** 1 State Key Laboratory of Oral Diseases, Sichuan University, Chengdu 610000, China; hanqi992011@163.com (Q.H.); libolei@stu.scu.edu.cn (B.L.); zhouxd@scu.edu.cn (X.Z.); 2016324035015@stu.scu.edu.cn (Y.G.); 2015324035041@stu.scu.edu.cn (S.W.); limingyun@scu.edu.cn (M.L.); renbiao@scu.edu.cn (B.R.); wanghaohao.aries@foxmail.com (H.W.); zalmancoco@163.com (K.Z.); pengx@scu.edu.cn (X.P.); fengmingye@gmail.com (M.F.); 2 Department of Operative Dentistry and Endodontics, West China Hospital of Stomatology, Sichuan University, Chengdu 610000, China; 3 Biomaterials & Tissue Engineering Division, Department of Endodontics, Periodontics and Prosthodontics, University of Maryland Dental School, Baltimore, MD 21201, USA; hxu@umaryland.edu (H.H.K.X.); MWeir@umaryland.edu (M.D.W.)

**Keywords:** alkyl chain group, QAMs, antibacterial, demineralization

## Abstract

The objectives of this study were to investigate the effects of dental adhesives containing quaternary ammonium methacrylates (QAMs) with different alkyl chain lengths (CL) on ecological caries prevention in vitro. Five QAMs were synthesized with a CL = 3, 6, 9, 12, and 16 and incorporated into adhesives. Micro-tensile bond strength and surface charge density were used to measure the physical properties of the adhesives. The proportion change in three-species biofilms consisting of *Streptococcus mutans, Streptococcus sanguinis*, and *Streptococcus gordonii* was tested using the TaqMan real-time polymerase chain reaction. Lactic acid assay, MTT [3-(4,5-dimethyl-thiazol-2-yl)-2,5-diphenyltetrazolium bromide] assay, exopolysaccharide staining, live/dead staining, scanning electron microscopy (SEM), and transverse microradiography (TMR) were performed to study the anti-biofilm and anti-demineralization effects of the dental adhesives. The results showed that incorporating QAMs with different alkyl chain lengths into the adhesives had no obvious effect on the dentin bond strength. The adhesives containing QAMs with a longer alkyl chain developed healthier biofilms. The surface charge density, anti-biofilm, and anti-demineralization effects of the adhesives increased with a CL of the QAMs from 3 to 12, but decreased slightly with a CL from 12 to 16. In conclusion, adhesives containing QAMs with a tailored chain length are promising for preventing secondary caries in an “ecological way”.

## 1. Introduction

Dental caries has emerged as a major public health problem across the world, affecting about 60–90% of schoolchildren and the vast majority of adults [1,2]. Resin composites are popular to fill tooth cavities by adhesive bonding [3,4,5]. Although great progress has been made in developing composites and adhesives, half of all restorations failed within 10 years, and replacing them cost 50–70% of the dentists’ time [6,7]. Secondary caries is the primary reason for this failure [8].

The cause of dental caries is the accumulation of dental biofilms, characterized by the accumulation of aciduric and acidophilic bacteria, which leads to the accumulation of acid within the biofilms and finally to demineralization on enamel [9,10]. Therefore, multiple strategies and anti-caries agents were developed to enhance the anti-biofilm capability of dental materials [11,12,13]. Quaternary ammonium methacrylates (QAMs) is a group of the antibacterial agents with a promising clinical value [14], including 12-methacryloyloxydodecylpyridinium bromide (MDPB) [15,16], quaternary ammonium dimethacrylate (QADM) [17,18], quaternary ammonium methacrylate polymer (QAMP) [19,20], 2-acryloxyethyltrimethylammonium chloride (ATA) [21,22], methacryloxyl ethyl cetyl dimethyl ammonium chloride (DMAE–CB) [23], quaternary ammonium polyethylenimine (PEI) [24], and dimethylaminododecyl methacrylate (DMADDM) [25,26,27]. 

According to the modern etiology of dental caries, it is advisable to control the oral microbiota at levels compatible with health, without killing the beneficial bacteria and losing the key benefits delivered by these resident microbes [28,29]. It is possible that some QAMs might be effective in preventing secondary caries in a more “ecological way”; hence, their ability to reduce the carious risk of dental plaque should be studied along with their “contact-killing effect”. However, only a few studies investigated how QAMs could change the proportions of different types of bacteria in dental biofilms [26].

Increasing the alkyl chain length (CL) could increase the hydrophobicity, which could enhance the propensity to penetrate the hydrophobic bacterial membrane. Some investigators, therefore, tried to synthesize QAMs with different chain lengths to study their anti-caries potential in different dental materials [30,31,32,33,34]. Previous studies have found that DMADDM (CL = 12) and DMAHDM (CL = 16) had a strong anti-biofilm effect on planktonic bacteria and microcosm biofilms [33,34,35]. However, no study compared the effect of QAMs with different chain lengths on regulating the ecological balance of multi-species biofilms and on inhibiting the demineralization on dental enamel. 

**Figure 1:**
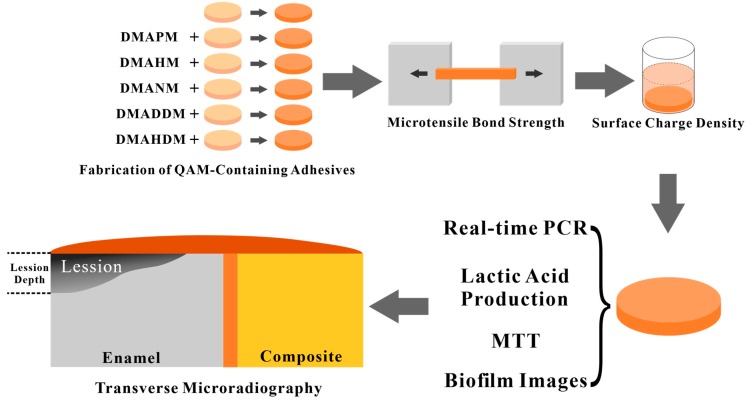
Design of the present study.

Therefore, the objectives of this study were to investigate the anti-biofilm effect of dental adhesives containing QAMs with different chain lengths (CL = 3, 6, 9, 12, and 16) on different types of bacteria in controlled multi-species biofilms, to compare their regulating effect on the biofilm developmental process and their anti-demineralization effect on dental enamel (Figure 1). It was hypothesized that: (1) QAMs with different chain lengths will not only influence the growth and metabolism of biofilm, but also regulate the proportion of bacteria in dental plaque; (2) adhesives containing QAMs with different chain lengths show different abilities in inhibiting the demineralization on dental enamel.

## 2. Results

### 2.1. The Physical Properties of Adhesives Containing QAMs

**Figure 2:**
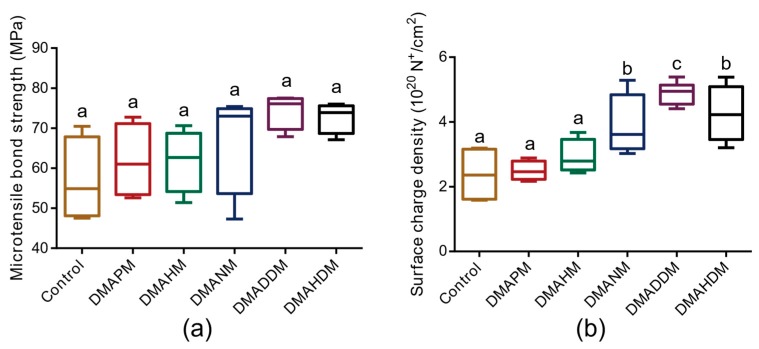
The physical properties of cured adhesives. (**a**) The micro-tensile bond strength (*n* = 4); and (**b**) the surface charge density (*n* = 12) of adhesives. Bars with the same letter indicate a value having no significant distance, and those with different letters indicate a significant difference (*p* < 0.05).

The micro-tensile bond strength of the adhesives is plotted in Figure 2a. The adhesives containing 5% dimethylaminopropyl methacrylate (DMAPM, CL = 3), 5% dimethylaminohexyl methacrylate (DMAHM, CL = 6), 5% dimethylaminononyl methacrylate (DMANM, CL = 9), 5% dimethylaminododecyl methacrylate (DMADDM, CL = 12), and 5% dimethylaminohexadecyl methacrylate (DMAHDM, CL = 16) were similar in bond strength (*p* > 0.05) to those of the control group. 

Figure 2b shows that the surface charge density of the adhesive disks containing QAMs with longer alkyl chain lengths (CL = 9, 12, and 16) was significantly higher than that of the control group (*p* < 0.05), that of the adhesive containing DMADDM being the highest (*p* < 0.05). Compared with the control group, the surface charge density of the adhesives containing DMAPM and DMAHM increased slightly (*p* > 0.05). In general, the surface charge density of the novel adhesives increased when the CL of QAMs was from 3 to 12.

### 2.2. The Microbial Composition of Three-Species Biofilms on the Adhesives

**Figure 3:**
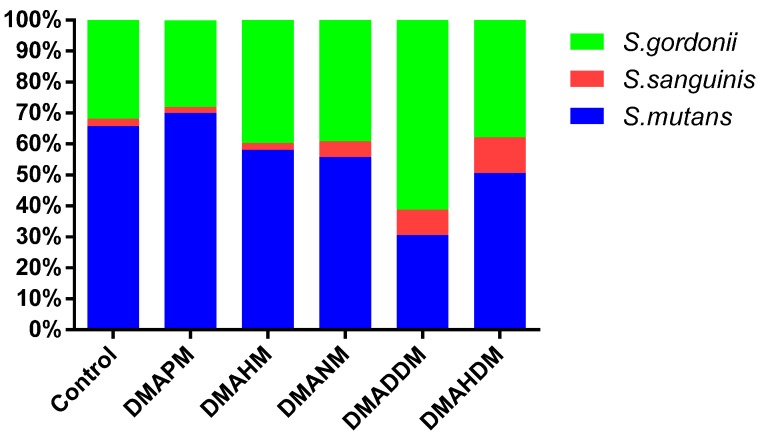
Microbial composition of three-species biofilms on adhesive disks, counted using the TaqMan real-time polymerase chain reaction.

The microbial composition of three-species biofilms on adhesive disks cultured for 24 h was tested using the real-time polymerase chain reaction (real-time PCR). Figure 3 displays the ratio variation of three bacteria in multi-species biofilms. Among the control group, the DMAPM and the DMAHM group, there was an increasing proportion of *S. mutans* in biofilms, and the percentage reached around 70%. Thus, the proportion of *S. sanguinis* and *S. gordonii* decreased. However, in the DMADDM group and the DMAHDM group, the ratio of *S. mutans* steadily decreased to 30% and 50%, respectively, and the ratio of *S. sanguinis* also decreased. As for *S. gordonii*, the ratio reached up to 8% and 11% in the DMADDM group and the DMAHDM group, respectively.

### 2.3. Metabolic Activity and Lactic Acid Production

**Figure 4:**
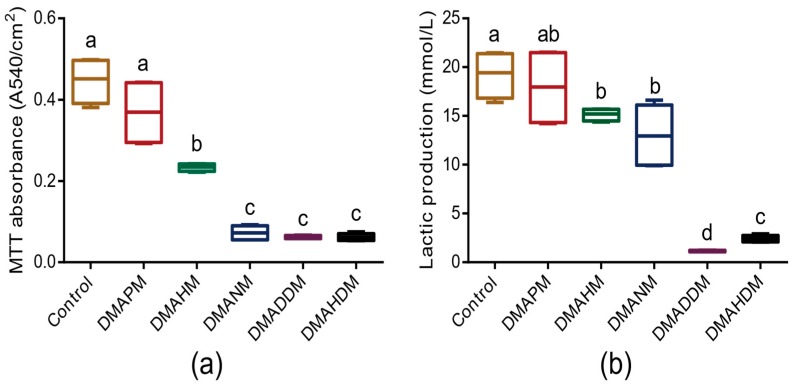
Metabolic activity and lactic acid-producing ability of three-species biofilms on adhesive disks. (**a**) MTT assay for the metabolic activity of three-species biofilms on the adhesive disks (*n* = 6); (**b**) the lactic acid produced by the biofilms (*n* = 6). Bars with the same letter indicate a value having no significant distance, and those with different letters indicate a significant difference (*p* < 0.05).

The metabolic activity result of the three-species biofilms measured using the MTT [3-(4,5-dimethyl-thiazol-2-yl)-2,5-diphenyltetrazolium bromide] assay is shown in Figure 4a. Compared with that of the control group, the metabolic activity of DMAHM, DMADNM, DMADDM, and DMAHDM groups decreased significantly (*p* < 0.05). In contrast, the DMAPM group’s metabolic activity decreased slightly (*p* > 0.05).

The lactic acid production of the biofilms on adhesive disks is plotted in Figure 4b. The lactic acid production of the DMAHM, DMADNM, DMADDM, and DMAHDM groups was significantly lower than that of the control group (*p* < 0.05). In particular, the biofilms on the adhesive disks containing DMADDM produced the least lactic acid. The DMAPM group was not significantly distinct from the control group in lactic acid production (*p* > 0.05).

### 2.4. Three-Species Biofilms Observed by Scanning Electron Microscopy and Confocal Laser Scanning Microscope

**Figure 5:**
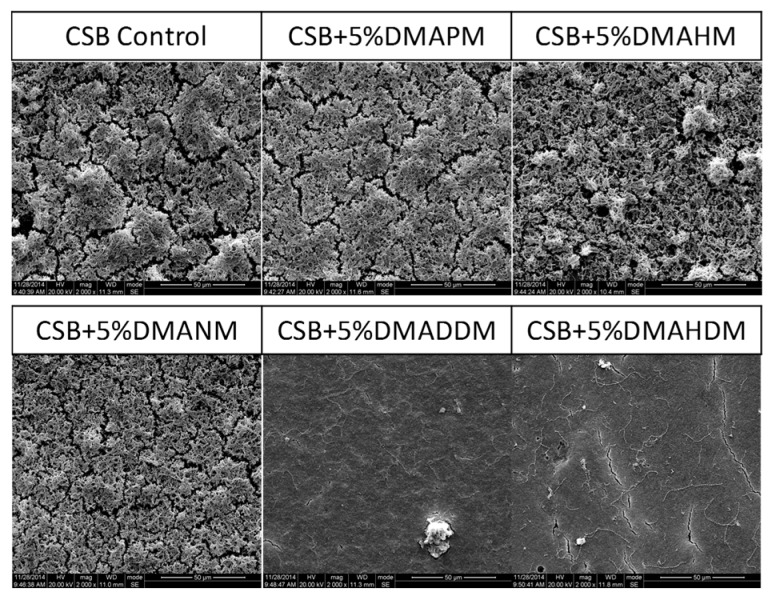
Scanning electron microscopy (SEM) micrographs of three-species biofilms on adhesive (Clearfil SE Bond) disks containing quaternary ammonium methacrylates (QAMs) with different alkyl chain lengths (CL) (CL = 3, 6, 9, 12, and 16) or none.

Figure 5 shows the SEM micrographs of the biofilms on adhesive disks. The control group had the maximum amount of biofilm growth. The biofilms did not fully develop on the adhesive disks containing QAMs with a longer alkyl chain length (CL = 12 and 16).

**Figure 6:**
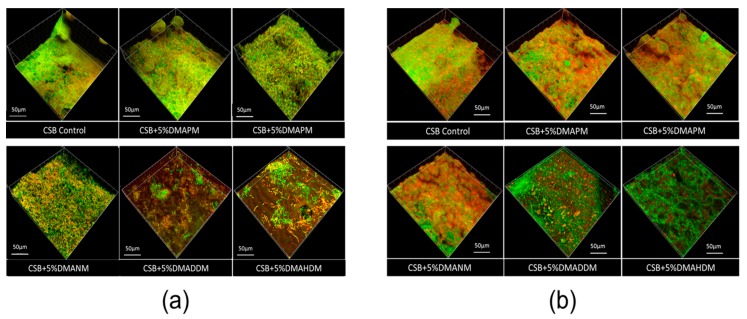
Confocal laser scanning microscope (CLSM) of three-species biofilms. (**a**) Live/dead staining of biofilms on the adhesive disks of the six groups. Bacterial cells were stained green, and dead cells were stained red; (**b**) exopolysaccharide (EPS) staining of biofilms on the adhesive disks of the six groups. Bacteria were stained green, and EPS was stained red.

The result of the live/dead bacteria staining is exhibited in Figure 6a. Biofilms on the adhesive disks containing DMAPM and no QAMs became denser than the biofilms on other adhesive disks. In contrast, the biofilms of the DMADDM and the DMAHDM group were hardly recognizable, indicating that these biofilms did not fully develop. Figure 6b shows the result of exopolysaccharide (EPS) staining. Red represents EPS staining, which can protect pathogenic bacteria and contribute to their pathogenicity. Here, too, the EPS staining of biofilms on the adhesives containing DMADDM and DMAHDM was hardly recognizable.

### 2.5. Anti-Demineralization Effect Tested by Transverse Microradiography

**Figure 7:**
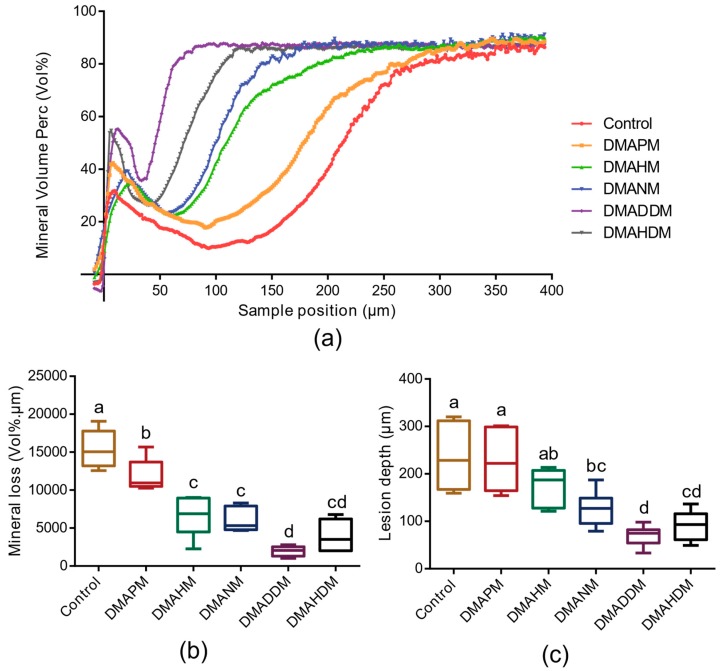
The enamel demineralization tested by transverse microradiography (TMR). (**a**) Mineral volume content of the enamel bonding with composites by QAM adhesives; (**b**) mineral loss (*n* = 6); and (**c**) lesion depth (*n* = 6) of enamel bonding with composites by adhesives containing QAMs with different alkyl chain lengths (CL = 3, 6, 9, 12, and 16) or none. Bars with the same letter indicate a value having no significant distance, and those with different letters indicate a significant difference (*p* < 0.05).

Figure 7a shows the mineral volume content of the enamel bonding with composites by QAM adhesives. Figure 7b,c show the mineral loss and lesion depth of the enamel bonding with composites by adhesives, respectively. Compared with that of the control group, the mineral loss of the groups containing QAMs decreased significantly (*p* < 0.05). The lesion depth of the DMADNM, DMADDM, and DMAHDM groups was significantly lower than that of the control group (*p* < 0.05). In particular, in both mineral loss and lesion depth, the DMADDM group was the lowest.

## 3. Discussion

To investigate bioactive materials, multiple biofilm models were used with their individual respective advantages in vitro, including the single-species biofilm model, the controlled multi-species biofilm model, and the saliva-derived microcosm biofilm model [36]. The controlled multi-species biofilm and saliva-derived microcosm biofilm models are often used to investigate ecological phenomena. However, compared with the saliva-derived microcosm biofilm model with a complex microbial community, the controlled multi-species biofilm model with a defined community has an advantage in achieving a high degree of reproducibility between experimental runs [36]. Thus, a three-species biofilm model used in a previous study [26] was chosen to investigate the effect of QAMs on the microbial community in the present study. 

The three-species biofilm consists of *S. mutans, S. gordonii*, and *S. sanguinis*, which were chosen for their presence in the oral cavity and their importance in the formation of dental plaque biofilm [26,37]. As an aciduric and acidophilic bacterium, *S. mutans* has been always associated with dental caries [38]. *S. gordonii* has been considered as an early colonizer of the dental plaque biofilm, while *S. sanguinis* has been linked with low caries risk as a common inhabitant [39]. Increasing the alkyl chain length (CL = 3, 6, 9 and 12) decreased the proportion of *S. mutans* and increased the proportion of *S. gordonii* in the biofilm, which partly influenced the metabolism of the biofilm and finally contributed to inhibiting the demineralization on dental enamel in an “ecological way”.

The antibacterial mechanism of QAMs has been thought to be that QAMs bind to bacteria, and finally cause bacterial lysis. When the negatively charged bacterial cells contact the positive quaternary amine charge (N+), the electric balance is disturbed and the bacteria explode under their own osmotic pressure. Like needles bursting balloons, long cationic polymers can penetrate the cells by disrupting the membranes [24,30]. Therefore, increasing the alkyl chain length [31,34], the density of adhesives was shown to decrease biomass more effectively. Zhou’s study [34] found that the charge density and the anti-biofilm effect of the adhesive containing QAMs with different chain lengths (CL = 6, 9, 12 and 16) increased with the chain length of the QAMs. The results of the present study in general correspond with those of Zhou’s study, except that our results show that the charge density and the anti-biofilm effect of the adhesive containing QAMs with a chain length of 12 were higher than those of the adhesive containing QAMs with a chain length of 16. The reason for this difference may be that we used Clearfil SE (CSE) Bond as the dissolution, instead of the Scotchbond Multi-Purpose used by Zhou et al. However, the effect of dissolutions on anti-caries adhesive still needs further investigation.

In conclusion, this study investigated the anti-caries effects of the CSE Bond adhesive with different alkyl chain length QAMs on controlled multi-species biofilms for the first time. CSE Bond adhesives containing DMADDM and DMAHDM shifted the composition of the multi-species biofilm and reduced the acid production, EPS synthesis, and mineral loss compared with the control group and other groups. Novel materials with tailored chain lengths are promising for use in dental adhesives, cements, sealants, and composites for preventing secondary caries through inhibiting the biofilms and the demineralization on dental enamel in an “ecological way”.

## 4. Materials and Methods

### 4.1. Synthesis of QAM-Containing Adhesives

QAMs with different CL were synthesized via the addition reaction of tertiary amines with organohalides [33,35]. To synthesize dimethylaminopropyl methacrylate (DMAPM, CL = 3, molecular weight: 168.2991), 10 mmol of DMAEMA, 10 mmol of 1-bromopropane (BPP) (TCI America, Portland, OR, USA), and 3 g of ethanol were added to a vial, which was capped and stirred at 70 °C for 24 h. After the reaction was completed, ethanol was evaporated. Then DMAPM was obtained as a clear liquid, which was verified via Fourier transform infrared spectroscopy. Another four groups of QAMs with CL = 6, 9, 12, and 16 were synthesized in this way [40], namely:

1. DMAEMA was reacted with 1-bromohexane to form dimethylaminohexyl methacrylate (DMAHM, CL = 6, molecular weight: 210.3788).
2. DMAEMA was reacted with 1-bromononane to form dimethylaminononyl methacrylate (DMANM, CL = 9, molecular weight: 252.4586).
3. DMAEMA was reacted with 1-bromododecane to form dimethylaminododecyl methacrylate (DMADDM, CL = 12, molecular weight: 294.5383).
4. DMAEMA was reacted with 1-bromohexadecane to form dimethylaminohexadecyl methacrylate (DMAHDM, CL = 16, molecular weight: 350.6446).

QAMs with CL = 3, 6, 9, 12, and 16 were incorporated into Clearfil SE (CSE) Bond (Kuraray Dental, Tokyo, Japan), which was used as the matrix bonding system, at a mass fraction of 5%, in accordance with previous studies.

We chose to light cure resin adhesives for 10 s. The light intensity (LK-G31, ZZlinker, Zhengzhou, China) of a curing unit can reach 1100 mW/cm^2^, and the wave length can be between 420 nm and 480 nm.

### 4.2. Specimen Preparation

The specimens for the biofilm experiments were prepared following a previous study [34,35,41]. Briefly, using the cover of a sterile 24-well plate (Costar, Corning Inc., Corning, New York, NY, USA) as a mold, composite disks were fabricated. An adhesive containing five QAMs and a control adhesive surrounded two sides of composite (CLEARFIL AP-X, Kuraray America, Pasadena, TX, USA, Shade: A3) disks and was light-cured for 10 s. The composite disks were immersed in distilled water for 24 h to remove unpolymerized monomer, and then they were sterilized in an ethylene oxide sterilizer (AnproleneAN 74i, Andersen, Haw River, NC, USA).

### 4.3. Micro-Tensile Bond Strength Test

A micro-tensile bond strength test was carried out in accordance with a recent study [25]. Briefly, this experimental study used extracted bovine teeth. The roots of the teeth were removed 2–3 mm below the cement–enamel junction by means of a water-cooled low-speed cutting saw (EMUC6/FC6, Leica Microsystems, Wetzlar, Germany). Then, a flat surface was prepared by removing the occlusal third of the tooth crowns with the cutting saw to expose mid-coronal dentin. The dentin surface was polished with 600-grit SiC paper to create a smear layer. The crown segments were randomly allocated to three groups. The dentin surface was applied over the primer by using a brush-tipped applicator, rubbed for 15 s and applied and light cured for 10 s (LK-G31, ZZlinker, Zhengzhou, China). Resin composite build-ups were constructed with 2-mm increments and light cured for 60 s. After storage in deionized water at 37 °C for 24 h, each tooth was vertically sectioned into 0.9-mm thick serial slabs using a cutting saw with water-cooling. The slabs were sectioned into 0.9 × 0.9 mm composite dentin beams, excluding those situated peripherally and showing the presence of enamel. The beams in six groups were immediately subjected to the micro-tensile bond strength test. Each beam was stressed to fracture under tension in a computer-controlled Universal Testing Machine (MTS, Eden Prairie, MN, USA) at a crosshead speed of 1 mm/min. The cross-sectional area at the site of fracture was measured with a pair of digital calipers (Fisher Scientific, Pittsburg, PA, USA) to calculate the micro-tensile bond strength. Samples were measured three times, and four replicates were tested for each group every time.

### 4.4. Surface Charge Density Test

The surface charge density of quaternary ammonium groups on the disk surface was quantified using a fluorescein dye method described previously. The disks were placed in a 24-well plate. Fluorescein sodium salt (200 μL of 10 mg/mL) in deionized (DI) water was added into each well, and the specimens were left for 10 min at room temperature in the dark. After removing the fluorescein solution and rinsing extensively with DI water, each sample was placed in a new well, and 200 μL of 0.1% (by mass) of cetyltrimethylammonium chloride (CTMAC) in DI water was added. The samples were shaken for 20 min at room temperature in the dark to absorb the bound dye. The CTMAC solution was supplemented with 10% (by volume) of 100 mM phosphate buffer at pH 8. The sample absorbance was read at 501 nm using a plate reader (SpectraMax M5, Molecular Devices, Sunnyvale, CA, USA). The fluorescein concentration was calculated using Beers Law and an extinction coefficient of 77 mM^−1^ cm^−1^. Using a ratio of 1:1 for fluorescein molecules to the accessible quaternary ammonium groups, the surface charge density was calculated as the total molecules of charge per exposed surface area. Twelve replicates were tested for each group.

### 4.5. Bacteria Incubation and Biofilm Formation

Multi-species biofilms were cultured in accordance with a previous study [26,41]. First, *Streptococcus mutans, Streptococcus gordonii*, and *Streptococcus sanguinis* were cultured anaerobically in a brain heart infusion broth (BHI; Difco, Sparks, MD, USA) at 37 °C (90% N_2_, 5% CO_2_, 5% H_2_). Cultures of *S. mutans* UA159, *S. gordonii* ATCC 10558, or *S. sanguinis* ATCC 10556 were regulated to 1 × 10^7^ CFUs/mL of suspension solution in BHI broth. The tests were repeated three times. For multi-species biofilm formation, bacterial suspensions were mixed to obtain an inoculum containing a defined microbial population consisting of *S. mutans* (10^7^ CFU/mL), *S. gordonii* (10^7^ CFU/mL), and *S. sanguinis* (10^7^ CFU/mL) in BHI with 1% sucrose. The medium used for biofilm development was exchanged every 24 h. The multi-species biofilms at 24 h were collected for the following experiments.

### 4.6. DNA Isolation and Real-Time PCR 

DNA isolation and real-time PCR were performed in accordance with a previous study [26,37]. The total DNA of the biofilms were isolated and purified using a TIANamp Bacteria DNA kit (TIANGEN, Beijing, China) according to the manufacturer’s instructions. The bacteria were lysed using enzymatic lysis buffer (20 mM Tris–HCl, pH 8.0; 2 mM sodium EDTA, and 1.2% Triton X-100) containing 25 mg/mL of lysozyme at 37 °C for 1.5 h. The purity and the concentration of the DNA were detected using a NanoDrop 2000 spectrophotometer (Thermo Scientific, Waltham, MA, USA). The extracts were stored at −20 °C. 

A TaqMan real-time polymerase chain reaction (PCR) (Life Technologies, Carlsbad, CA, USA) was performed to quantify the absolute quantification for *S. mutans, S. gordonii*, and *S. sanguinis* using a Bio-Rad CFX96 TM Real-time System (Bio-Rad Laboratories, Hercules, CA, USA). Each real-time PCR reaction mix contained 10 μL of TaqMan Universal PCR Premix Ex Taq, 1.5 μL of template, and 250 nM (each) of sense and antisense primer, and 250 nM of TaqMan probe was placed in each well, using the following cycling conditions: 95 °C for 3 min, followed by 40 cycles of 95 °C for 10 s, and 56 °C for 30 s. The fluorescence was detected after each cycle. The specificity of the probes was confirmed by conventional PCR, and the standard curves of these bacteria were plotted for each primer/probe set by using threshold cycle values obtained by amplifying successive 10-fold dilutions of known concentrations of DNA, which stands for the corresponding concentration of bacteria from 10^9^ CFUs to 10^4^ CFUs. The quantifications of the three strains were calculated based on standard curves generated using the respective standard strains. Three replicates were tested for each group.

### 4.7. The MTT Assay and Lactic Acid Measurement

Disks with 24-h biofilms were rinsed in cysteine peptone water (CPW) to remove loose bacteria. CPW was filtered using a cylinder membrane filter. Each disk was placed in a new 24-well plate with 1.5 mL of buffered peptone water (BPW) supplemented with 0.2% sucrose. The biofilms remained stable after culturing for 3 h in the BPW medium, which had a relatively high buffer capacity. The biofilms were incubated at 5% CO_2_ and 37 °C for 3 h to produce acid. The BPW solutions were stored for lactate analysis. The lactate concentrations in the BPW solutions were determined using an enzymatic (lactate dehydrogenase) method. A microplate reader (SpectraMax M5, (Molecular Devices, Sunnyvale, CA, USA)) was used to measure the absorbance at 340 nm (optical density OD_340_) prior to and after collecting the BPW. Standard curves were prepared using a standard lactic acid (Supelco Analytical, Bellefonte, PA, USA).

The MTT [3-(4,5-dimethyl-thiazol-2-yl)-2,5-diphenyltetrazolium bromide] assay is a colorimetric assay for assessing three-species metabolic activity, which measures the enzymatic reduction of MTT (a yellow tetrazole) to formazan and is used to test the bacterial viability and bacterial reproductive capacity of biofilm on the disks [37,41]. Each disk was transferred to a new 24-well plate, to which the MTT solution (0.5 mg/mL MTT in PBS) was added, and incubated at 37 °C in 5% CO_2_ for 1 h. During this process, metabolically active bacteria metabolized the MTT and reduced it to purple formazan inside the living cells. After 1 h, the disks were transferred to a new 24-well plate, 1 mL of dimethyl sulfoxide (DMSO) was added to solubilize the formazan crystals, and the plate was incubated for 20 min with gentle mixing at room temperature. After brief mixing via pipetting, 200 μL of the DMSO solution from each well was transferred to a 96-well plate, and the absorbance at 540 nm (OD_540_) was measured via the microplate reader. A higher absorbance indicated a higher formazan concentration, which in turn indicated more metabolic activity in the biofilm present on the composite disk. Six replicates were tested for each group.

### 4.8. Biofilm Images

Scanning electron microscopy (SEM), live/dead bacteria staining, and exopolysaccharides (EPS) staining were used to examine structural and morphological changes of the biofilms.

For the SEM images, the 24-h biofilms on the disks were rinsed with PBS and then immersed in 1% glutaraldehyde in PBS for 4 h at 4 °C. Then, the specimens were subjected to graded ethanol dehydrations and rinsed twice with 100% hexamethyldisilazane. The specimens were then sputter-coated with gold and examined using scanning electron microscopy (SEM, Quanta 200, FEI, Hillsboro, OR, USA).

For live/dead bacteria staining, the 24-h biofilms on the disks were washed three times with PBS and then stained using the BacLight live/dead bacterial viability kit (Molecular Probes, Eugene, OR, USA). Live bacteria were stained with Syto 9 to produce green fluorescence, and bacteria with compromised membranes were stained with propidium iodide to produce a red fluorescence. The disks were examined using a confocal laser scanning microscope (CLSM) (Leica, Wetzlar, Germany). An EPS assay was conducted in accordance with previous studies. Briefly, the bacterial cells were labeled by using 2.5 μM SYTO 9 green fluorescent nucleic acid stain (480/500 nm; Molecular Probes Inc., Eugene, OR, USA). The polysaccharides were labeled with 2.5 μM Alexa Fluor 647-dextran conjugate (molecular weight, 10,000; maximum absorbance wavelength, 647 nm; maximum fluorescence emission wavelength, 668 nm; Molecular Probes Inc., Eugene, OR, USA). Then, the 24-h biofilms on the disks were examined using a CLSM (Leica, Wetzlar, Germany). All 3-dimensional reconstructions of the biofilms were performed with Imaris 7.0.0 (Bitplane, Zürich, Switzerland), and the quantification of the live/dead and EPS bacteria volume ratio was performed with Image-Pro Plus (Media Cybernetics, Silver Spring, MD, USA) and COMSTAT.

### 4.9. Transverse Microradiography

This experimental study was performed on extracted bovine teeth. The labial dental crown was cut into 5 um × 5 um × 4 um. The parallel dentin surfaces were measured using the Rockwell hardness test [41] (Zwick, Ulm, Germany). Bovine specimens in the range from 350 KHN to 550 KHN were collected for the demineralization test.

The specimens were formed into approximately 8 mm × 5 mm × 4 mm. Each sealed specimen was immersed in distilled water for 24 h to remove unpolymerized monomer. Both the samples and the 24-well plate were sterilized in an ethylene oxide sterilizer (AnproleneAN 74i, Andersen, Haw River, NC, USA) and enclosed at room temperature.

Controlled oral multi-species biofilms were cultured in accordance with a previous study [26]. An amount of 2 mL bacterial suspension solution was added to each well with a fixed bovine sample, which was cultured anaerobically in a brain heart infusion medium with 1% sucrose at 37 °C (90% N_2_, 5% CO_2_, 5% H_2_). The medium refreshed every 12 h. After 3 days of biofilm demineralization treatment for bovine with antibacterial adhesive, samples were taken out and rinsed softly with distilled water three times to remove the biofilms [42]. Six replicates were tested for each group.

After the oral bacterial biofilm demineralization treatment, all specimens were cut again and polished by hand plane—parallel from both sides with water-cooled silicon carbide disks (800-, 1200-grade papers, ANSI grit; Buehler, Lake Bluff, IL, USA) to a thickness ranging around 150 nm. Micrographs were taken of each section together with an aluminum calibration step wedge with 14 steps. Kodak high-resolution plates (type 1A) were used with a 15-min exposure using a CuKa X-ray source (Philips B.V., Amsterdam, The Netherlands) operating at 25 kV and 10 mA. The film–focus–specimen distance was 40 cm in height. The plates were developed using Kodak brand materials and following the manufacturers’ instructions. The developed film was analyzed using a transmitted light microscope with a ×20 objective (Axioplan; Zeiss, Oberkochen, Germany), equipped with a CCD camera (Canon, Tokyo, Japan) connected to a PC (TOSHIBA, Tokyo, Japan) with frame grabber, data acquisition, and calculation software (TMR 2012, Inspektor Research BV, Amsterdam, The Netherlands). The lesion depth was calculated using a threshold of 90% of the mineral content of sound dentine (i.e., 47.5%). The integrated mineral loss (vol. % mm, DZ) and the lesion depth (mm) were also calculated, using a two-stage analysis procedure.

### 4.10. Statistical Analysis

The Mann–Whitney U-test was performed to detect the significant effects of the variables at a *p* value of 0.05. The SPSS21.0 (SPSS Inc., Chicago, IL, USA) software was used for the statistical analysis.
